# Bioinformatics proficiency among African students

**DOI:** 10.3389/fbinf.2024.1328714

**Published:** 2024-06-20

**Authors:** Ashraf Akintayo Akintola, Abdullahi Tunde Aborode, Muhammed Taofiq Hamza, Augustine Amakiri, Benjamin Moore, Suliat Abdulai, Oluyinka Ajibola Iyiola, Lateef Adegboyega Sulaimon, Effiong Effiong, Adedeji Ogunyemi, Boluwatife Dosunmu, Abdulkadir Yusif Maigoro, Opeyemi Lawal, Kayode Raheem, Ui Wook Hwang

**Affiliations:** ^1^ School of Industrial Technology Advances, Kyungpook National University, Daegu, Republic of Korea; ^2^ NOBLEKINMAT Ltd. Bioinformatics Research Group, Ibadan, Nigeria; ^3^ Department of Chemistry, Mississippi State University, Starkville, MS, United States; ^4^ Green Climate Fund, Incheon, Republic of Korea; ^5^ ProCogia, Vancouver, BC, Canada; ^6^ European Molecular Biology Laboratory - European Bioinformatics Institute, Wellcome Genome Campus, Cambridgeshire, United Kingdom; ^7^ Department of Biochemistry, Fountain University, Osogbo, Nigeria; ^8^ Department of Zoology, University of Ilorin, Ilorin, Nigeria; ^9^ Department of Biochemistry, Crescent University, Abeokuta, Nigeria; ^10^ Department of Medical Laboratory Sciences, Babcock University, Ilishan-Remo, Nigeria; ^11^ Center for Biotechnology and Genomics, Texas Tech University, Lubbock, TX, United States; ^12^ University of Kansas, Lawrence, KS, United States; ^13^ Department of Life Sciences, College of Life Sciences and Bioengineering, Incheon National University, Incheon, Republic of Korea; ^14^ Department of Food Science, University of Guelph, Guelph, ON, Canada; ^15^ Cancer Research Artificial Intelligence (CARESAI), Hobart, Australia; ^16^ Department of Biology, Teachers College and Institute for Phylogenomics and Evolution, Kyungpook National University, Daegu, Republic of Korea; ^17^ Institute for Korean Herb-Bio Convergence Promotion, Kyungpook National University, Daegu, Republic of Korea

**Keywords:** bioinformatics, literacy, Africa, students, training

## Abstract

Bioinformatics, the interdisciplinary field that combines biology, computer science, and data analysis, plays a pivotal role in advancing our understanding of life sciences. In the African context, where the diversity of biological resources and healthcare challenges is substantial, fostering bioinformatics literacy and proficiency among students is important. This perspective provides an overview of the state of bioinformatics literacy among African students, highlighting the significance, challenges, and potential solutions in addressing this critical educational gap. It proposes various strategies to enhance bioinformatics literacy among African students. These include expanding educational resources, fostering collaboration between institutions, and engaging students in research projects. By addressing the current challenges and implementing comprehensive strategies, African students can harness the power of bioinformatics to contribute to innovative solutions in healthcare, agriculture, and biodiversity conservation, ultimately advancing the continent’s scientific capabilities and improving the quality of life for her people. In conclusion, promoting bioinformatics literacy among African students is imperative for the continent’s scientific development and advancing frontiers of biological research.

## 1 Introduction

Bioinformatics is a rapidly evolving interdisciplinary field that merges biology, computer science, and data analysis to understand, interpret, and manage biological data. It plays a crucial role in advancing our understanding of genetics, genomics, and personalized medicine. However, the level of bioinformatics literacy among African students is a matter of concern (Mulder et al., 2016; Aron et al., 2017). Despite the growing importance of bioinformatics in modern biology and the potential it holds for addressing health, agriculture, and environmental challenges on the continent, many African students face significant barriers to gaining proficiency in this field (Akintola et al., 2022a; 2022b).

African countries are characterized by a rich biodiversity and a diverse range of health-related issues, thus, making bioinformatics an essential tool for research and development (Karikari, 2015; Wonkam, 2021). Unfortunately, limited access to resources, educational infrastructure, and opportunities for training and research in bioinformatics can hinder African students from fully harnessing the potential of this field.

This topic is of importance as it not only addresses a knowledge gap but also has significant implications for the future of science and technology on the continent. Developing bioinformatics literacy among African students can contribute to advancements in healthcare, agriculture, and other areas, ultimately helping to address some of the pressing challenges faced by African countries.

In this perspective, we explore the current state of bioinformatics literacy among African students, the challenges they face, and potential solutions to bridge the gap. We examine the role of education, training programs, and international collaborations in promoting bioinformatics literacy in Africa. Additionally, we discuss the efforts that Noblekinmat Ltd-an impact development organization with the mandate of bioinformatics training and consultancy in Africa, has been making to improve bioinformatics education and research on the continent.

## 2 Current state of bioinformatics literacy among African students

The current state of bioinformatics literacy among African students reflects both opportunities and challenges. While there have been significant efforts to promote bioinformatics education and research on the continent, there are still notable gaps in terms of access to resources and training.

### 2.1 Issues and opportunities

Access to up-to-date bioinformatics resources, scientific journals, textbooks, software, and databases are perennial issues that would constantly be discussed regarding bioinformatics education in the continent. Many bioinformatics software and tools are proprietary and expensive. The lack of financial means to purchase licenses for these tools limits their practice and learning. These limitations thus hinder the ability to engage in cutting-edge research in the continent. Poor internet connectivity and a lack of high-performance computing facilities which is still rife especially in sub-Saharan Africa also impede bioinformatics research and learning opportunities for students (Ras et al., 2021). Downloading large data sets and accessing free online courses or tutorials which are essential for bioinformatics education also becomes a problem when engaging in bioinformatics training. This is further compounded by the epileptic power supply in most parts of the continent.

As an impact development company with the mandate of providing top-tier bioinformatics training and consultancy services in Africa, NOBLEKINMAT LTD. has been organizing several bioinformatics trainings in which the authors have been resource persons at trainings, seminars, and webinars in different aspects of bioinformatics (Table 1). Poor and/or expensive internet services, low-performance computers (based on most student’s budgets), and unstable power supply are the main impediments to getting lots of students on board for most of the training. It also becomes a herculean task to retain lots of those who have been trained previously. Thus, it becomes a perennial problem if the impending challenges are not addressed.

**TABLE 1:** Summary of bioinformatics training organized by NOBLEKINMAT Ltd.

S/N	Training theme	Date	Registered participant	Location
*1*	Bioinformatics 1.0 webinar on DNA and Molecular Data analysis	February 27, 2021	300	Virtual
*2*	Bioinformatics 2.0 webinar on how to analyze DNA and RNA sequences	31 July 2021	258	Virtual
*3*	ENSEMBL genome browser training	24 February 2022	1,008	Virtual
*4*	Bioinformatics Seminar- Bioinformatics of Cancer and Infectious Diseases	5 March 2022	150	Virtual
*5*	Bioinformatics 3.0 webinar (Genomics)	March 12–18, 2022	1,008	Virtual
*6*	Genomics training (1)	6 May 2022	208	Virtual
*7*	Genomics training (2)	16–19 June 2022	196	Virtual
*8*	Bioinformatics 4.0 webinar	1–8 August 2022	250	Virtual
*9*	Population Genetics training	3 October 2022	50	Virtual
*10*	Bioinformatics training tour (Including ENSEMBL genome browser training)	26–15 January February 2023	300	Hybrid (Onsite and Virtual)
*11*	Bioinformatics (for beginners) Webinar	March 31 – 1 April 2023	40	Virtual
*12*	Bioinformatics Mini Course	August 2 – 31 October 2023	48	Virtual
*13*	Bioinformatic For You (Batch 1)	30 October 2023	50	Virtual
*14*	ENSEMBL genome browser training	12 January 2024	250	Virtual
*15*	UNIPRO GENE training	January 11 & 13, 2024	100	Virtual
*16*	Bioinformatics For you (Batch 2)	January 2–15, 2024	45	Virtual

Consequently, the previously highlighted problems have a pressing effect on the number of trained students in the field which also translates to the low number of trained instructors. Getting the instructors to deliver virtual lectures is no longer a problem as there are hundreds of free bioinformatics resources available online. However, the paucity of experienced bioinformatics educators in African institutions remains a significant barrier. Quality training relies on knowledgeable instructors who can guide students effectively (Munung et al., 2021).

**FIGURE 1:**
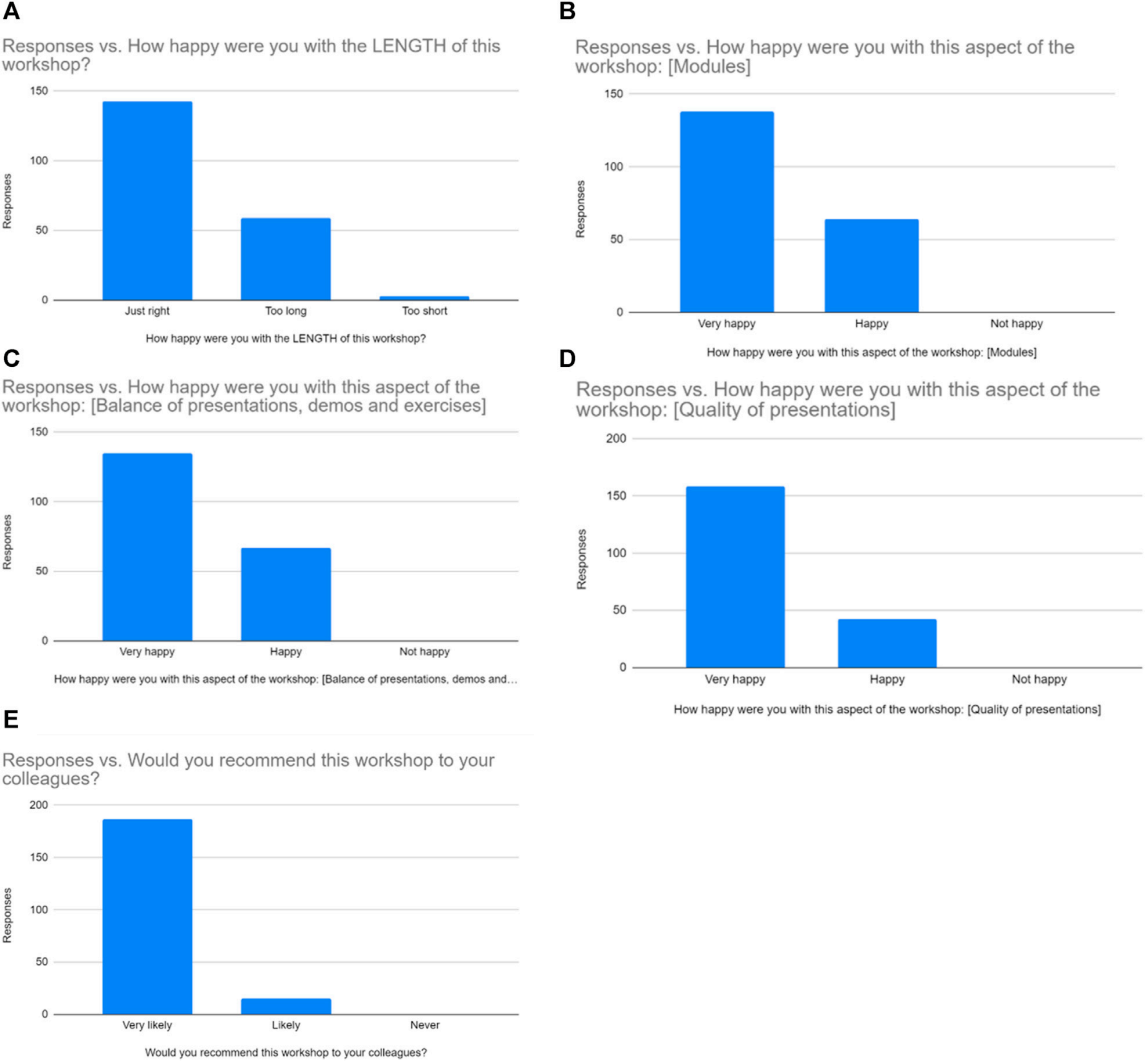
Responses of participants when asked how happy they were with the **(A)** Length **(B)** Modules **(C)** Balance of presentations, demos, and exercises **(D)** Quality of presentation, and **(E)** if they would recommend the workshop to their colleagues.

In addition, collaborative efforts between African universities and international institutions would ensure progress in training students and researchers in bioinformatics. These partnerships are vital for improving bioinformatics literacy (Mulder et al., 2016). For example, between January to February 2023, NOBLEKINMAT LTD conducted a bioinformatics training tour in collaboration with the European Bioinformatics Institute, UK, and the Institute for Phylogenomics and Evolution, Kyungpook National University, South Korea, across four Universities in Nigeria. Resource persons from these institutions conducted a blend of virtual and onsite bioinformatics training for participants in four Nigerian Universities. These universities include the University of Ilorin, Babcock University, Fountain University, and Crescent University. The training had in attendance several participants from more than eleven universities, polytechnics, as well as research institutes. As part of the ENSEMBL genome browser training, a survey was also conducted to ascertain the satisfaction of the participants in terms of modules being taught, the balance and quality of presentations, demonstrations, and exercises given as well as their views on the length of the workshop and willingness to recommend such trainings to their colleagues (Figure 1). The responses were an indication of the success of the program.

This success story is also based on the efforts of initiatives like H3ABioNet which have been established to develop sustainable bioinformatics capacity in Africa, offering training and resources to students and researchers (Mulder et al., 2016). The African BioGenome Project is another effort recording massive success in advancing bioinformatics education in the continent (Ebenezer et al., 2022). The Open Institute of the African BioGenome Project aims to bridge the gap in African biodiversity genomics and bioinformatics. African governments and non-governmental organizations are also gradually recognizing the importance of bioinformatics and investing in educational and research programs. These initiatives are helping to bridge the literacy gap (Jongeneel et al., 2017). On September 4, 2023, the African Centers for Disease Control and Prevention (Africa CDC) also launched its first bioinformatics training workshop which is aimed at expanding valuable skillsets and genomics capacity of the African continent (Africa CDC, 2023).

Furthermore, the African Society of Human Genetics (AfSHG) as well as the African Society for Bioinformatics and Computational Biology have been contributing to expanding the bioinformatics capacity on the continent through trainings and Omics Codeathon (ASBCB, 2023) aimed at students and researchers from the continent.

## 3 Present challenges and proffered solutions

As earlier posited, the development of bioinformatics proficiency within African universities is hindered by various obstacles. However, there exist viable strategies to overcome these challenges and facilitate its advancement. Bioinformatics is an indispensable tool in the progress of biomedical research as it facilitates the examination and comprehension of intricate biological information (Akintola et al., 2022a). African universities that possess bioinformatics proficiency have the potential to enhance healthcare outcomes and tackle the distinct health obstacles that are widespread in the region, including infectious diseases, neglected tropical diseases, and genetic disorders (Akintola et al., 2022a).

Insufficient computational infrastructure and resources pose a challenge to bioinformatics research in numerous institutions in Africa (Rotimi et al., 2017). The essential components encompassed in this are advanced computing systems with superior capabilities, dependable access to internet connectivity, stable electricity (Laurance et al., 2015), and financial resources to procure requisite software and databases. One potential solution to address this challenge is to establish partnerships between African and international institutions, which can facilitate access to computational resources and training programs (Ojo and Omabe, 2011). The provision of bioinformatics infrastructure in universities can be facilitated by governmental bodies and funding agencies.

The African continent is currently experiencing a dearth of proficient bioinformatics trainers and researchers (Nembaware and Mulder, 2019). The insufficiency of qualified personnel poses a constraint on the accessibility of bioinformatics courses and the provision of guidance to students (Giovanni et al., 2023). A potential solution to this issue is to facilitate faculty exchange programs and training initiatives, whereby seasoned bioinformaticians from different geographical locations can conduct workshops and training sessions at African universities. This can be augmented by online courses and resources that are available to a wider demographic.

The absence of specialized bioinformatics curricula is a prevalent issue in African universities, where comprehensive programs or courses that cater to the specific requirements of the region are not readily available (Ojo and Omabe, 2011). The incorporation of bioinformatics into current life sciences curricula and the establishment of specialized bioinformatics programs can effectively mitigate this deficiency (Karikari et al., 2015; TastanBishop et al., 2015). The establishment of partnerships between academic institutions and bioinformatics research organizations can serve to advance the creation of pertinent educational programs and foster the dissemination of information. Conferences, workshops, and seminars can serve as effective means to augment researchers' cognizance regarding the significance of bioinformatics in their respective domains (Akintola et al., 2022a). The establishment of regional and international collaborations can facilitate the exchange of knowledge and the development of capacity.

The accessibility and quality of data are fundamental aspects of conducting bioinformatics research, particularly in the biological domain (Anderson et al., 2007). Notwithstanding, data accessibility and quality in certain African nations may be restricted due to factors such as data-sharing policies, data management practices, and insufficient research funding (Hamdi et al., 2021). The promotion of open data-sharing policies, advocacy for data standards, and provision of support for local data generation initiatives can contribute to the enhancement of data accessibility and quality.

The retention of bioinformatics talent in Africa is a concern, as there is a potential for brain drain, whereby proficient bioinformatics professionals may pursue career opportunities outside the continent due to restricted prospects (Isewon et al., 2022). Limited opportunities, resource constraints, migration for education and career prospects, and recognition are some of the several factors that contribute to the brain drain in bioinformatics in Africa (Isewon et al., 2022). To address this issue, it is crucial to establish a conducive setting that nurtures professional growth and scholarly prospects within the locality (Mboowa et al., 2021). To retain talent, it is recommended to provide competitive salaries and better incentives. Offering competitive salaries, research grants, and recognition for contributions made within the continent can incentivize professionals to stay and contribute to the local bioinformatics landscape.

Establishing research networks and encouraging collaborations between African research institutions can facilitate knowledge exchange, skills development, and resource-sharing. Africa Biogenome project is another key example of this (Ebenezer et al., 2022). The roles of policy and government support are also key in transforming bioinformatics education in the continent (Hamdi et al., 2021). By implementing policies that prioritize research and innovation, allocate sufficient funds to support scientific endeavours, and offer incentives for professionals to stay and contribute.

While the challenges persist, there is a growing recognition of the importance of bioinformatics in Africa. International collaborations, success stories, and the emergence of training programs are positive signs that are contributing to improved bioinformatics literacy among African students. However, sustained efforts and investments are needed to ensure that students across the continent can fully harness the potential of this field.

It is well known that African countries varied not only geographically, but also economically. This has an impact on their respective system of education, and life balance which are attributes to research and development. For example, in a systematic review performed on microbiome-related research, the results show that more research is performed from strong economic countries such as South Africa, Egypt, and Nigeria as compared to poor countries such as Niger, and Mauritania (Maigoro et al., 2023). This can be related to their living standard as well as their literacy level. Added to that, some institutions of learning fill the literacy gap through workshops and special training. Fatumo demonstrates a systematic way of enhancing learning to supplement higher learning (Fatumo et al., 2014). This is performed with the help of Regional Student Groups (RSGs) especially in the emerging fields of bioinformatics and computational biology. A similar effort was made by the RSGs subset group from South Africa called the South African Bioinformatics Student Council (SASBiSC). They not only guide students on bioinformatics skills but also engage them in potential job prospects (Rafael et al., 2017).

## 4 Potentials of bioinformatics proficiency among African students

Bioinformatics holds a lot of potential to transform different sectors of the African landscape. The agricultural sector, for example holds significant importance in numerous African nations. The field of bioinformatics has the potential to contribute significantly to the enhancement of crops, the development of disease-resistant varieties, and the improvement of livestock breeding programs (Xue et al., 2008; Weckwerth, 2011). The incorporation of bioinformatics into agricultural research by African universities has the potential to bolster food security, foster sustainable farming methods, and augment agricultural output.

Africa is renowned for its abundant biodiversity, encompassing a variety of ecosystems, fauna, and flora. The continent is blessed with a wide range of plant and animal species (Ebenezer et al., 2022). The field of bioinformatics has the potential to aid in the comprehension and preservation of this distinct biodiversity. African academic institutions possessing bioinformatics proficiency have the potential to make valuable contributions to the domains of conservation, species identification, and natural resource management.

The genetic diversity of African populations presents an opportunity to gain insights into diseases, drug responses, and personalized medicine approaches through the study of their genetic variations (Owolabi et al., 2023). The utilization of bioinformatics tools and analyses is crucial in the interpretation of genetic information and the comprehension of the genetic underpinnings of various diseases (Gurwitz et al., 2017). African academic institutions possessing bioinformatics proficiency have the potential to make valuable contributions to genomics research and healthcare customized to the indigenous populace (Gurwitz et al., 2017).

The promotion of bioinformatics literacy in African universities facilitates capacity building and skill development, thereby fostering the development of a proficient workforce within the region (Nembaware and Mulder, 2019; Mboowa et al., 2021). This facilitates the ability of African researchers and scientists to autonomously perform bioinformatics analyses, participate in interdisciplinary partnerships, and make contributions to worldwide scientific progress. Proficiency in bioinformatics can also lead to prospects for job placement and business ventures in the expanding realm of genomics and data-centric life sciences (Prost et al., 2020).

International collaborations and knowledge exchange are essential for the advancement of bioinformatics, which is a field with a global reach (Hamdi et al., 2021; Owolabi et al., 2023). African universities that possess bioinformatics proficiency have the potential to engage in international partnerships, exchange knowledge and information, and make valuable contributions to worldwide scientific pursuits (TastanBishop et al., 2015). This initiative fosters the incorporation of African perspectives into global bioinformatics research, while also promoting inclusivity and diversity.

The optimal path to progress entails a multifaceted strategy that encompasses the participation of various stakeholders, such as academic institutions, governmental bodies, funding entities, global organizations, and the bioinformatics sector, working together collaboratively. Through the adoption of strategic initiatives aimed at addressing these challenges, African universities can enhance bioinformatics literacy and make significant contributions to the progress of genomics, proteomics, drug discovery, and other domains of biological research.

In conclusion, the significance of bioinformatics proficiency in African educational institutions lies in its potential to propel progress in various domains such as biomedical research, agricultural growth, biodiversity preservation, personalized medicine, skill enhancement, and global partnerships. When achieved, it would confer authority to African researchers, and in essence, it would foster progress in scientific research to tackle challenges specific to the region in healthcare, agriculture, and conservation.

## Data Availability

The original contributions presented in the study are included in the article/Supplementary material, further inquiries can be directed to the corresponding authors.

## References

[B1] African CDC (2023). First bioinformatics training workshop kicks off at the Africa CDC state-of-the-art laboratory and training center. Available at: https://africacdc.org/news-item/first-bioinformatics-training-workshop-kicks-off-at-the-africa-cdc-state-of-the-art-laboratory-and-training-centre/on (Accessed December 27, 2023).

[B2] Akintola A. A. Aborode T. A. Hwang U. W. (2022a). Africa needs genomic epidemiologists - correspondence. Int. J. Surg. 108, 106999. 10.1016/j.ijsu.2022.106999 36356825

[B3] Akintola A. A. Hwang U. W. Aborode A. T. (2022b). Africa needs more bioinformaticians for population studies. Nature 605 (7911), 619. 10.1038/d41586-022-01378-8 35610376

[B4] Anderson N. R. Lee E. S. Brockenbrough J. S. Minie M. E. Fuller S. Brinkley J. (2007). Issues in biomedical research data management and analysis: needs and barriers. J. Am. Med. Inf. Assoc. 14, 478–488. 10.1197/jamia.m2114 PMC2244904 17460139

[B5] Aron S. Gurwitz K. Panji S. Mulder N. Consortium H. (2017). H3ABioNet: developing sustainable bioinformatics capacity in Africa. EMBnet.J. 23, e886. 10.14806/ej.23.0.886

[B6] ASBCB (2023). ASBCB Omics Codeathon 2023. Available at: https://www.asbcb.org/eventsonDecember.

[B7] Ebenezer T. E. Muigai A. W. T. Nouala S. Badaoui B. Blaxter M. Buddie A. G. (2022). Africa: sequence 100,000 species to safeguard biodiversity. Nature 603 (7901), 388–392. 10.1038/d41586-022-00712-4 35292740

[B8] Fatumo S. Shome S. Macintyre G. (2014). Workshops: a great way to enhance and supplement a degree. PLoS Comput. Biol. 10 (2), e1003497. 10.1371/journal.pcbi.1003497 24586140 PMC3937119

[B9] Giovanni M. Y. Whalen C. Hurt D. E. Ware-Allen L. Noble K. McCarthy M. (2023). African Centers of excellence in bioinformatics and data intensive science: building capacity for enhancing data intensive infectious diseases research in Africa. J. Infect. Dis. Microbiol. 1 (1), 006. 10.37191/mapsci-jidm-1(2)-006 37987019 PMC10658664

[B10] Gurwitz K. T. Aron S. Panji S. Maslamoney S. Fernandes P. L. Judge D. P. (2017). H3ABioNet Consortium's Education Training and Working Group as members of the H3Africa Consortium. Designing a course model for distance-based online bioinformatics training in Africa: the H3ABioNet experience. PLoS Comput. Biol. 13 (10), e1005715. 10.1371/journal.pcbi.1005715 28981516 PMC5628786

[B11] Hamdi Y. Zass L. Othman H. Radouani F. Allali I. Hanachi M. (2021). Human OMICs and computational biology research in Africa: current challenges and prospects. OMICS A J. Integr. Biol. 25 (4), 213–233. 10.1089/omi.2021.0004 PMC8060717 33794662

[B12] Isewon I. Soremekun C. Adebiyi M. Adetunji C. Ogunleye A. J. Bajeh A. O. (2022). Strengthening bioinformatics and genomics analyses skills in Africa for attainment of the sustainable development goals: report of the 2nd conference of the Nigerian bioinformatics and genomics network. Am. J. Trop. Med. Hyg. 16, 21–23. 10.4269/ajtmh.21-1164 PMC9294681 35576945

[B13] Jongeneel C. V. Achinike-Oduaran O. Adebiyi E. Adebiyi M. Adeyemi S. Akanle B. (2017). Assessing computational genomics skills: our experience in the H3ABioNet African bioinformatics network. PLoS Comput. Biol. 13 (6), e1005419. 10.1371/journal.pcbi.1005419 28570565 PMC5453403

[B14] Karikari T. K. (2015). Bioinformatics in Africa: the rise of Ghana? PLoS Comput. Biol. 17 (9), e1004308. 10.1371/journal.pcbi.1004308 PMC4574930 26378921

[B15] Karikari T. K. Quansah E. Mohamed W. M. Y. (2015). Developing expertise in bioinformatics for biomedical research in Africa. Appl. Transl. Genom 6, 31–34. 10.1016/j.atg.2015.10.002 26767162 PMC4699396

[B16] Laurance W. F. Sloan S. Weng L. Sayer J. A. (2015). Estimating the environmental costs of africa’s massive ‘‘Development corridors. Curr. Biol. 25, 3202–3208. 10.1016/j.cub.2015.10.046 26628009

[B17] Maigoro A. Y. Muhammad M. Bello B. Useh U. Lee S. (2023). Exploration of gut microbiome research in Africa: a scoping review. Journ. Med. Food 26 (9), 616–623. 10.1089/jmf.2023.k.0005 37523293

[B18] Mboowa G. Sserwadda I. Aruhomukama D. (2021). Genomics and bioinformatics capacity in Africa: no continent is left behind. Genome 64 (5), 503–513. 10.1139/gen-2020-0013 33433259 PMC12179689

[B19] Mulder N. J. Adebiyi E. Alami R. Benkahla A. Brandful J. Doumbia S. (2016). H3ABioNet, a sustainable pan-African bioinformatics network for human heredity and health in Africa. Genome Res. 26 (2), 271–277. 10.1101/gr.196295.115 26627985 PMC4728379

[B20] Munung N. S. de Vries J. Pratt B. (2021). Genomics governance: advancing justice, fairness and equity through the lens of the African communitarian ethic of Ubuntu. Med. Health Care Philos. 24 (3), 377–388. 10.1007/s11019-021-10012-9 33797712 PMC8349790

[B21] Nembaware V. Mulder N. (2019). The african genomic medicine training initiative (AGMT): showcasing a community and framework driven genomic medicine training for nurses in Africa. Front. Genet. 10, 1209. 10.3389/fgene.2019.01209 31921282 PMC6934054

[B22] Ojo O. O. Omabe M. (2011). Incorporating bioinformatics into biological science education in Nigeria: prospects and challenges. e Educ. Niger. prospects challenges. Infect Genet Evol 11 (4), 784–787. 10.1016/j.meegid.2010.11.015 21145989

[B23] Owolabi P. Adam Y. Adebiyi E. (2023). Personalizing medicine in Africa: current state, progress and challenges. Front. Genet. 19 (14), 1233338. 10.3389/fgene.2023.1233338 PMC10546210 37795248

[B24] Prost S. Winter S. De Raad J. Coimbra R. T. F. Wolf M. Nilsson M. A. (2020). Education in the genomics era: generating high-quality genome assemblies in university courses. Gigascience 1 (6), giaa058. 10.1093/gigascience/giaa058 PMC7268781 32491162

[B25] Rafael C. N. Ambler J. Niehaus A. Ross J. Bishop O. T. (2017). Establishment of “the South African bioinformatics student Council” and activity highlights. EMBnet. J. 23, 903. 10.14806/ej.23.0.903

[B26] Ras V. Carvajal-López P. Gopalasingam P. Matimba A. Chauke P. A. Mulder N. (2021). Challenges and considerations for delivering bioinformatics training in LMICs: perspectives from pan-african and Latin American bioinformatics networks. Front. Educ. 6, 710971. 10.3389/feduc.2021.710971

[B27] Rotimi C. N. Bentley A. R. Doumatey A. P. Chen G. Shriner D. Adeyemo A. (2017). The genomic landscape of African pop-ulations in health and disease. Hum. Mol. Genet. 26, R225–R236. 10.1093/hmg/ddx253 28977439 PMC6075021

[B28] Tastan Bishop O. Adebiyi E. F. Alzohairy A. M. Everett D. Ghedira K. Ghouila A. (2015). Bioinformatics education—perspectives and challenges out of Africa. Brief. Bioinform 16, 355–364. 10.1093/bib/bbu022 24990350 PMC4364068

[B29] Weckwerth W. (2011). Green systems biology—from single genomes, proteomes and metabolomes to ecosystems research and biotechnology. J. Proteomics 75 (1), 284–305. 10.1016/j.jprot.2011.07.010 21802534

[B30] Wonkam A. (2021). Sequence three million genomes across Africa. Nature 590 (7845), 209–211. 10.1038/d41586-021-00313-7 33568829 PMC9979155

[B31] Xue J. Zhao S. Liang Y. Hou C. Wang J. (2008). “Bioinformatics and its applications in agriculture,” in Computer and computing technologies in agriculture 2 Editor Li D. 977–982.

